# Domestic violence against pregnant women is a potential risk factor for low birthweight in full-term neonates: A population-based retrospective cohort study

**DOI:** 10.1371/journal.pone.0279469

**Published:** 2022-12-22

**Authors:** Ching-Heng Lin, Wei-Szu Lin, Hsiu-Yuan Chang, Shiow-Ing Wu

**Affiliations:** 1 Department of Medical Research, Taichung Veterans General Hospital, Taichung, Taiwan; 2 Department of Health Care Management, National Taipei University of Nursing and Health Sciences, Taipei, Taiwan; 3 Department of Public Health, College of Medicine, Fu Jen Catholic University, New Taipei City, Taiwan; 4 Department of Industrial Engineering and Enterprise Information, Tunghai University, Taichung, Taiwan; 5 Institute of Public Health and Community Medicine Research Center, National Yang-Ming University, Taipei, Taiwan; 6 Department of Protective Services, Ministry of Health and Welfare, Taipei, Taiwan; 7 Institute of Population Health Sciences, National Health Research Institutes, Zhunan, Taiwan; University of South Florida, UNITED STATES

## Abstract

Domestic violence’s most frequently reported outcomes are preterm delivery and low birthweight, both of which are the strongest correlates of mortality and morbidity. Several studies have shown that pregnant women with domestic violence during pregnancy were more likely to deliver low-birthweight and preterm neonates. However, there has been no consensus on associations between domestic violence and low-birthweight and preterm delivery. To examine the impact of domestic violence on birthweight stratified by preterm or full-term delivery, a population-based retrospective cohort study was conducted that linked four national databases in Taiwan. A total of 1,322 subjects associated with a report of domestic violence during pregnancy were compared with 485,981 subjects without any record of reported domestic violence. The percentage of low birthweight in the group exposed to domestic violence was significantly higher than in the unexposed group with full-term delivery (4.9% vs. 3.3%, p = 0.001). Multivariable logistic regression analysis showed that pregnant women exposed to domestic violence had an OR of 1.37 (95% CI 1.05, 1.79) for low birthweight in full-term delivery. However, domestic violence was not significantly associated with low birthweight in preterm delivery. Screening for intimate partner violence in the perinatal health care system should be seen as especially important for women who have had full-term low-birthweight neonates.

## Introduction

Violence against women is a worldwide public health issue and a violation of women’s human rights. Estimates published by the World Health Organization indicate that globally about 30% of women have been subjected to physical and/or sexual intimate partner violence (IPV) or to non-partner sexual violence [1].

IPV during pregnancy has been found to be associated with fatal and non-fatal adverse health outcomes for pregnant women and their babies. Fatal outcomes from violence during pregnancy are homicide or suicide [2]. Non-fatal outcomes include negative impacts on health behavior, pregnancy/birth outcomes, and physical and mental health [3]. The factors associated with negative health behavior include alcohol [4, 5], drug abuse [4, 6–8], smoking [7], and delayed prenatal care [7]. The factors associated with negative pregnancy and birth outcomes include low birthweight (LBW) [4–11], preterm labor/delivery [5, 12, 13], insufficient weight gain [6, 14], obstetric complications [15], sexually transmitted infections/human immunodeficiency virus [16], miscarriages [5], and unsafe abortions [4]. The factors associated with negative physical and mental health include injury [17], physical impairment, and depression [2, 4]. Although the impact of IPV is multifactorial, most of the related factors are highly preventable and can be identified through screening during the perinatal period [18].

Among the above-mentioned factors, there is considerable literature on the effects of IPV on LBW and preterm deliveries. IPV’s most frequently reported outcomes are preterm delivery and LBW [19], both of which are the strongest correlates of mortality and morbidity among neonates [20]. However, no consensus on the relationship between these three variables (LBW, preterm delivery, and violence) has been reached. For the association between IPV during pregnancy and LBW, many studies found positive relationships [4–10, 12, 21–25] and others found no relationship [2, 26–32]. For the association between IPV and preterm delivery, most studies found a positive association [4, 5, 9, 11, 12, 25, 33], while others found no significant difference [17, 29, 32, 34]. Thus, additional clarification of the relationship between these variables could prove desirable.

A report cautioned that LBW has so many interacting factors that it is very important to distinguish between preterm and full-term deliveries when attempting to sort out the complex interrelations of factors affecting birthweight [35]. In addition, due to the complex, close correlation between LBW and preterm delivery—and the fact that both of them could be indicative of violence—it is better to use stratified analysis by preterm or full-term delivery to identify the association between domestic violence and LBW.

However, very few studies have created categories such as preterm/LBW, preterm/normal birthweight (NBW), full-term/LBW, and full-term/NBW to identify their associations with violence among pregnant women. Some studies—for instance Coker et al. [36], Cokkinides et al. [17], and Campbell et al. [30]—examined this issue with stratified analysis by preterm/full-term delivery and normal/LBW, but their results are quite different. Coker et al. found that women delivering preterm/LBW and term/LBW infants were more likely to report abuse during pregnancy [36]; Cokkinides et al. found that physical violence was associated only with a risk of preterm NBW [17]; and Campbell et al. did not find a significant effect of abuse on LBW in full-term or preterm babies in multivariable analysis [30]. The inconsistency of these results on the possible effects of violence during pregnancy on LBW-preterm complex might be due to limitations of the studies, such as in their design (e.g., case–control and cross-sectional studies) or by having only a small sample size.

The aim of this study is to conduct a retrospective population-based cohort study with a large sample size from Taiwan’s Domestic Violence Dataset and to elucidate whether the risk of LBW, stratified by preterm and term deliveries, was different among women who, respectively, did or did not experience domestic violence during pregnancy.

## Materials and methods

### Study sample

Our research team linked and analyzed four national datasets, all of which are under the supervision of the Ministry of Health and Welfare, Taiwan; reporting to these datasets is mandatory. After the government agencies anonymized the records, the data were made available to the current research team in 2018, as detailed in an earlier report [37]. Those datasets were as follows: (1) the 2004–2014 Taiwan Maternal and Child Health Database, in which parent and child IDs are linked together; (2) the National Health Insurance Research Database (NHIRD), which is derived from the National Health Insurance Administration and includes 99% of the total population’s medical care claims data since 1995 [38]; (3) the Birth Notification System (BNS) from 2001 to 2016, which contains reports for all neonates weighing more than 500 g or with a gestational age greater than 20 weeks [39]; and (4) the Domestic Violence Dataset from 2011 to 2016, which is built from mandatory reports to the government under article 50 of the Domestic Violence Prevention Act [40]. Different datasets provided different periods of coverage, with the overlapping timeframe from 2011 to 2014. Women with pregnancy from 2011 to 2013 were our target population, with figures from 2014 included to ensure coverage of all women who were pregnant in 2013.

**Fig 1:**
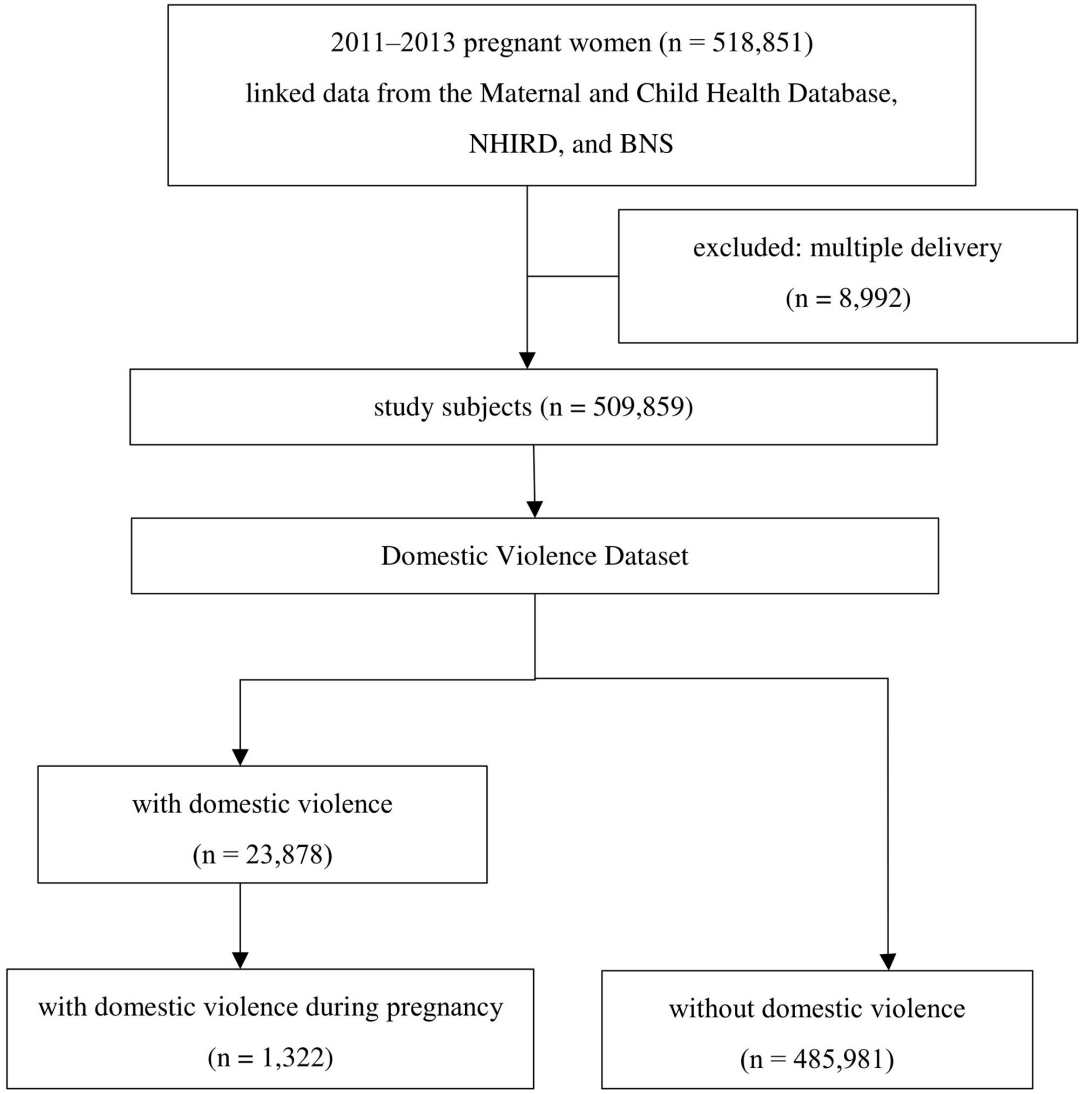
Flow diagram of the study subjects.

In this retrospective cohort study, 518,851 women who were pregnant for their first childbirth were selected in the first step to link the Taiwan Maternal and Child Health Database with the NHIRD; the resulting dataset was then linked with the BNS from 2011 to 2013. After excluding 8,992 multiple deliveries (because such deliveries highly correlate with relatively low birthweight), the remaining 509,859 study subjects were linked with the Domestic Violence Dataset. A total of 23,878 subjects had been reported to have experienced some kind of domestic violence, and 485,981 subjects were without any report of domestic violence. A total of 1,322 (5.5%) of the 23,878 subjects experienced domestic violence prior to delivery but during pregnancy (Fig 1).

### Measures

#### Exposure information

Domestic violence was defined as an act of harassment or control, a threat, or another illegal action conducted against any family member that is physical, psychological, or economic in nature. In Taiwan, medical personnel, social workers, educational personnel, daily-life guidance personnel, police, immigration officers, etc., are required to report suspicions of domestic violence to the local competent authority within 24 hours; any cases that are evaluated as unconfirmed are subsequently removed from the dataset [40].

#### Outcome information

Birthweight was retrieved from birth certificates in the BNS, for which weight was measured using a digital scale and recorded in grams [39]. The definitions of LBW (as a weight of less than 2500 g, irrespective of gestational age) and preterm delivery (as babies born alive before 37 weeks of pregnancy) follow those of the World Health Organization [41].

#### Confounding variable information

Information regarding urbanization level of residence, income, maternal comorbidities, and pregnancy-related complications were retrieved from the NHIRD; and data regarding gestational age (≥ 37 weeks, < 37 weeks) and mode of delivery (vaginal, caesarean) were retrieved from the BNS. Having maternal comorbidities (diabetes mellitus, hypertension, or hyperlipidemia) was defined as having had an inpatient or outpatient diagnosis of those diseases during the two years before pregnancy. Having pregnancy-related complications (gestational diabetes mellitus, gestational hypertension, pre-eclampsia, placenta previa, or abruptio placenta) was defined having had an inpatient or outpatient diagnosis of those-conditions during pregnancy. Income was categorized into four levels, following the payroll bracket table published by the Ministry of Health and Welfare’s National Health Insurance Administration, and was recorded in Taiwan dollars [42].

### Statistical analysis

Data are presented as numbers (percentages) for categorical variables; and Pearson’s χ^2^ test for categorical variables was used for analysis. Multivariable logistic regression analyses were used to determine the impact of domestic violence on the risk of LBW stratified by preterm or full-term delivery, as shown by having an odds ratio with a 95% confidence interval (CI). All data were analyzed using SAS statistical software version 9.4 (SAS Institute, Cary, NC, USA). A p-value < 0.05 was considered to indicate statistical significance.

This study was approved by the Institutional Review Board of the National Health Research Institutes, Taiwan (IRB number: EC 1070601-E). All personal data obtained were anonymized before analysis, and informed consent was thus waived.

## Results

Table 1 shows the basic characteristics of the study population, with 1,322 subjects in the domestic violence exposed group and 485,981 in the unexposed group. Higher percentages of the domestic violence exposed group had a significantly lower maternal age (p < 0.001), had a lower income (p < 0.001), and lived in regions of lower urbanization (p < 0.001). The prevalences of hypertension (2.1% vs 1.0%, p < 0.001) and gestational hypertension (1.4% vs 0.7%, p = 0.002) were significantly higher among the violence-exposed group than the unexposed group, while gestational diabetes mellitus was significantly lower in the exposed group than the unexposed group (1.5% vs 2.5%, p = 0.024).

**Table 1:** Characteristics of study subjects.

Variables	Domestic violence exposed	Unexposed	Total	P-value
	(n = 1,322)	(n = 485,981)		
	n (%)	n (%)	n	
Maternal age				< 0.001
< 25	260 (19.7)	46128 (9.5)	46388	
25–34	810 (61.3)	345005 (71.0)	345815	
≥ 35	252 (19.1)	94848 (19.5)	95100	
Monthly income (NTD)				< 0.001
≤ 15840	488 (36.9)	54726 (11.3)	55214	
15841–28800	648 (49.0)	250792 (51.6)	251440	
28801–45800	121 (9.2)	116888 (24.1)	117009	
> 45800	65 (4.9)	63575 (13.0)	63640	
Urbanization of residence				< 0.001
Urban	752 (56.9)	302744 (62.3)	303496	
Suburban	192 (14.5)	59733 (12.3)	59925	
Rural	378 (28.6)	123495 (25.4)	123873	
Mode of delivery				0.052
Vaginal delivery	829 (62.7)	317110 (65.3)	317939	
Caesarean section	493 (37.3)	168871 (34.7)	169364	
Maternal comorbidity				
Diabetes mellitus	17 (1.3)	4313 (0.9)	4330	0.123
Hypertension	28 (2.1)	4619 (1.0)	4647	< 0.001
Hyperlipidemia	28 (2.1)	7593 (1.6)	7621	0.104
Pregnancy-related complication				
Gestational diabetes mellitus	20 (1.5)	12048 (2.5)	12068	0.024
Gestational hypertension	18 (1.4)	3225 (0.7)	3243	0.002
Pre-eclampsia or eclampsia	22 (1.7)	7112 (1.5)	7134	0.544
Placenta previa and abruptio placentae	55 (4.2)	18065 (3.7)	18120	0.395

**Fig 2:**
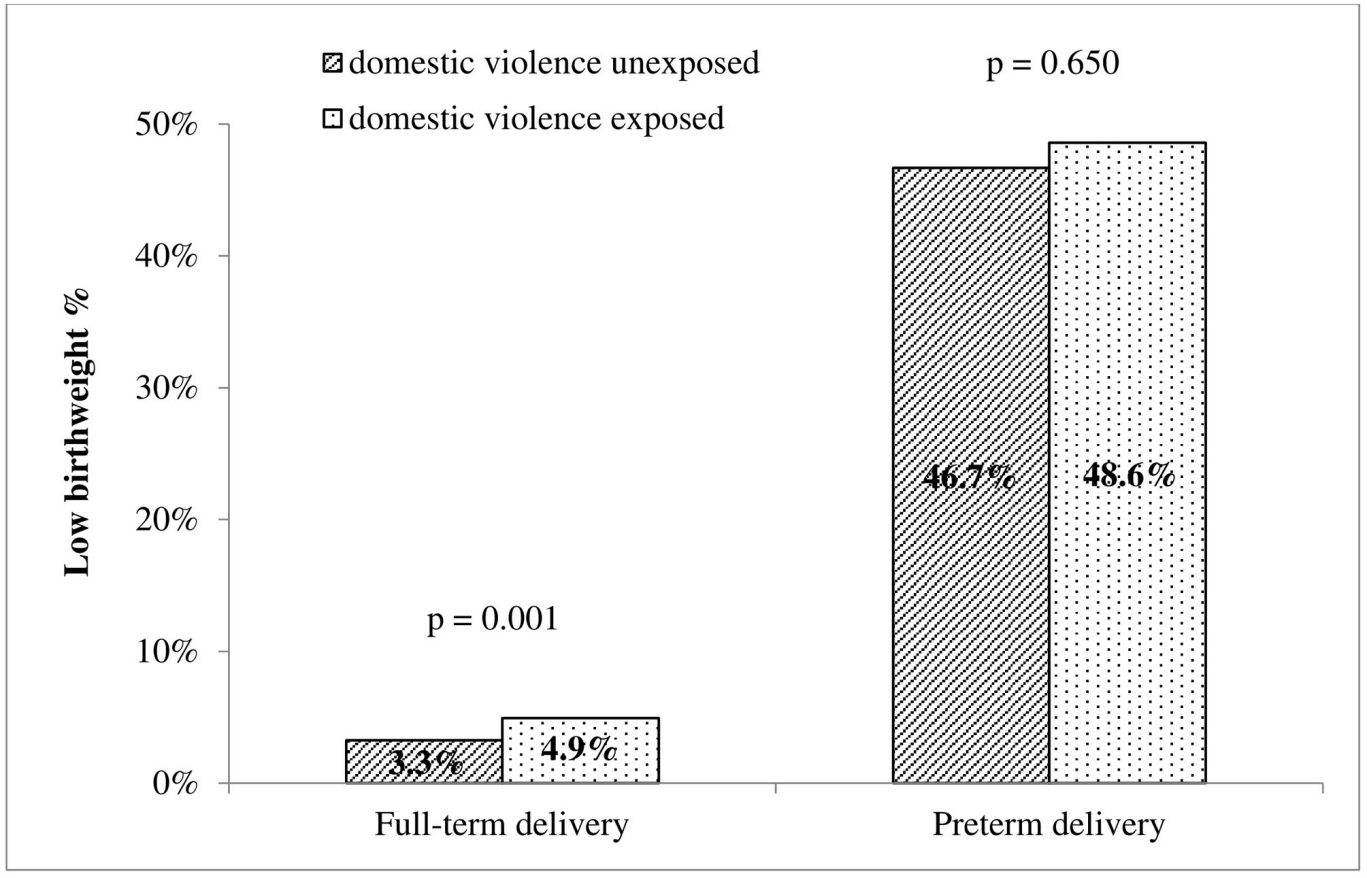
Percentage of low birthweight by domestic violence status and preterm/full-term delivery.

Fig 2 shows that the percentage of LBW in the domestic violence exposed group was significantly higher than in the unexposed group in full-term delivery (4.9% vs 3.3%, p = 0.001), while there was no statistically significant difference noted between the domestic violence exposed and unexposed groups in preterm delivery.

To adjust for potentially confounding effects from maternal age, income, urbanization of residence, mode of delivery, maternal comorbidity, and pregnancy-related complications, multivariable logistic regression analyses of factors associated with LBW were performed, stratified by full-term or preterm delivery. The results showed that pregnant women exposed to domestic violence had an OR of 1.37 (95% CI 1.05, 1.79) for LBW in full-term delivery. Domestic violence was not statistically significantly associated with LBW if babies had preterm delivery (Table 2). Other confounding effects significantly associated with LBW among preterm delivery were maternal age, income, mode of delivery, diabetes mellitus, hypertension, and variables of pregnancy-related complications (gestational diabetes mellitus, gestational hypertension, pre-eclampsia/eclampsia, placenta previa, and abruptio placentae).

**Table 2:** Multivariable analysis of factors associated with low birthweight (< 2500 g), stratified by full-term or preterm delivery.

Variable	Full-term delivery (≥ 37 weeks)			Preterm delivery (< 37 weeks)		
	OR	95% CI		OR	95% CI	
Violence exposed	1.37	1.05	1.79	1.04	0.74	1.47
Maternal age						
< 25	1.00(reference)			1.00(reference)		
25–34	0.77	0.73	0.81	0.82	0.76	0.89
≥ 35	0.78	0.73	0.83	0.78	0.71	0.85
Monthly income (NTD)						
≤ 15840	1.00(reference)			1.00(reference)		
15841–28800	0.84	0.80	0.89	0.86	0.81	0.92
28801–45800	0.79	0.75	0.84	0.98	0.91	1.06
> 45800	0.72	0.67	0.77	0.94	0.86	1.02
Urbanization of residence						
Urban	1.00(reference)			1.00(reference)		
Suburban	1.01	0.96	1.07	0.94	0.88	1.01
Rural	1.04	1.00	1.08	0.95	0.90	1.00
Mode of delivery						
Vaginal delivery	1.00(reference)			1.00(reference)		
Caesarean section	1.02	0.99	1.06	0.91	0.87	0.95
Maternal comorbidity						
Diabetes mellitus	0.70	0.57	0.85	0.49	0.41	0.58
Hypertension	1.75	1.52	2.00	1.20	1.04	1.38
Hyperlipidemia	0.98	0.85	1.12	0.86	0.74	1.00
Pregnancy-related complication						
Gestational diabetes mellitus	0.93	0.83	1.03	0.81	0.72	0.91
Gestational hypertension	2.94	2.58	3.34	2.09	1.73	2.54
Pre-eclampsia or eclampsia	5.58	5.14	6.07	5.54	4.99	6.15
Placenta previa and abruptio placentae	1.57	1.45	1.70	1.79	1.67	1.92

## Discussion

Because preterm delivery is intimately tied with LBW, much of the research in domestic violence has used multivariable analysis to control for preterm delivery and birthweight simultaneously, paying less attention to using stratification analysis to clarify their complex relationship. The most notable result to emerge from our study is that domestic violence is a risk factor for LBW among full-term delivery neonates after adjustment in multivariable analysis for maternal age, income, urbanization of residence, mode of delivery, maternal comorbidities, and pregnancy-related complications.

To our knowledge, this is the first population-based retrospective cohort study to investigate the association of domestic violence and LBW in Taiwan. The main strengths of our study are that we used a large national population-based database with a minimal risk of selection bias and good external validity to apply its results to the entire population of pregnant women.

Some of our findings differ from those of previous studies in this area. A cross-sectional study by Coker et al. of 274 women who reported IPV during a pregnancy found that when compared with term/NBW deliveries, women delivering preterm/LBW and term/LBW infants were more likely to report abuse during pregnancy, but there was no significant difference for preterm/NBW infants [36]. Cokkinides et al. conducted a population-based study of 6,143 women, 680 of whom reported physical violence within the 12 months of delivering a live infant; the study found that physical violence was associated with only risk of preterm NBW but not significantly associated with risk of preterm-LBW or term-LBW when compared with term-NBW [17]. Campbell et al. conducted a case–control study of 352 full-term infants and 326 preterm infants and in multivariable analysis did not find reported physical abuse to have a significant effect on LBW in full-term or preterm babies [30]. Although our results differ considerably from the above-mentioned studies, it could nevertheless be argued that our study’s population-based retrospective cohort design with more than 500,000 study subjects offers advantages. The earlier studies mentioned some limitations of their own. For example, a cross-sectional study (e.g., Coker et al.) may be subject to recall bias, and women experiencing abuse may have been more likely to report an adverse pregnancy outcome [36]. Campbell et al. also mentioned the disadvantage of recall bias in their case–control study [30]. In addition, even a population-based study with cross-sectional design (e.g., Cokkinides) may preclude examining physical violence as an antecedent factor to some outcomes and be subject to underreporting of violent experiences during pregnancy [17].

Our study did not find that domestic violence is a risk factor for LBW in preterm delivery; this might be because several other risk factors are more influential than domestic violence for LBW in preterm babies. Risk factors such as having delivered preterm before, being pregnant with multiple gestations, and having certain abnormalities of the reproductive organs were noted by the National Institutes of Health of the U.S. Department of Health and Human Services [43]. Certain medical conditions’ risk factors (including high blood pressure, placenta previa, abruptio placentae, diabetes, gestational diabetes, and rupture of the uterus) and some other risk factors (such as age of the mother, lifestyle factors, late or no health care during pregnancy, and domestic violence) also place women at higher risk for preterm labor and delivery [43]. Our multivariable logistic regression analyses showed that maternal age, low income, caesarean section, diabetes, hypertension, gestational diabetes mellitus, pre-eclampsia/eclampsia, and placenta previa/abruptio placentae were significantly associated with LBW in preterm delivery. That means the impacts from the above-mentioned risk factors were more influential than domestic violence for LBW in preterm neonates; but in those neonates with full-term delivery, domestic violence was statistically significantly associated with LBW after adjusting for the confounding factors.

The connection of violence with LBW might occur through both direct and indirect pathways [10]. The direct mechanism mainly involves physical trauma to the abdomen of pregnant women; and the indirect mechanism might be through an environment that contains factors such as abuse, low socioeconomic status, stress, inadequate prenatal care, or lack of social support [10, 44–46]. Domestic violence during pregnancy is a potential risk factor for LBW [22]; pregnant women with domestic violence may not present any symptoms and may attempt to conceal what they experienced at home [47]. Primary health care practitioners and clinicians are gatekeepers in screening for and identifying women exposed to violence; however, most screening practices are conducted only when women present obvious warning signs. Our findings suggest that perinatal health care system screening for IPV should be regarded as especially important for women who had a full-term neonate with LBW. This should be done in conjunction with other strategies, such as raising the awareness of and the screening capacity for IPV among primary health care workers, and revising the Maternal Health Booklet to remind obstetricians and gynecologists to complete and submit a Domestic Violence Screening and Assessment Form if a pregnant woman has any unexplained bruise.

### Limitations

Our research may have a few limitations. First, we are not able to focus on the different types of domestic violence (i.e., physical, emotional, and economic violence) because government regulations forbid the release of such information. However, a systematic review published in 2020 noted that using even undifferentiated IPV to examine associations with perinatal health can produce papers of high methodological quality [19]. The second limitation is that we cannot rule out some potentially confounding lifestyle behaviors, such as cigarette smoking and illicit drug use; information on these was not available in our four national databases. Ideally, these factors should be adjusted in multivariable analyses; however, the smoking and illicit drug use rates in women in Taiwan are quite low—around 2.4% and 1.3%, respectively—compared with those of Western countries, rendering the factors relatively inconsequential in the Taiwan population [48, 49]. The third limitation is that the underreporting of domestic violence is inevitable, given that many women fear retaliation by their intimate partners. Although the prevalence of domestic violence in our study is underestimated, the association between domestic violence and LBW in full-term delivery is statistically significant.

## Conclusions

This population-based retrospective cohort study has gone some way toward enhancing our understanding that domestic violence could be a risk factor for LBW when pregnant women are in full-term delivery. Our research might be useful in the possible revision of the perinatal health system’s screening for violence. Further work needs to be carried out to focus on the association between different types of violence (i.e., physical, emotional, and economic violence) and LBW, to establish a real-world mechanism for IPV screening in order to lower the adverse effects of domestic violence upon maternal and child health.
